# Alteration of gut microbiota in migraine patients with irritable bowel syndrome in a Chinese Han population

**DOI:** 10.3389/fneur.2022.899056

**Published:** 2022-11-16

**Authors:** Jieqiong Liu, Wenjing Tang, Lei Hou, Jing Wang, Rongfei Wang, Yaofen Zhang, Zhao Dong, Ruozhuo Liu, Shengyuan Yu

**Affiliations:** ^1^Department of Neurology, The First Medical Center, Chinese PLA General Hospital, Chinese PLA Medical School, Beijing, China; ^2^Department of Neurology, Cangzhou Central Hospital, Hebei Medical University, Cangzhou, China

**Keywords:** migraine, irritable bowel syndrome, gut microbiota, 16S rRNA, gut-brain axis

## Abstract

**Objective:**

Migraine is frequently reported in patients with irritable bowel syndrome (IBS), and emerging evidence suggests that gut microbiota plays a role in migraine and IBS. However, alterations in the gut microbiome in migraine patients with IBS remain unknown. This study aimed to explore the compositions of gut microbiota in migraine patients with IBS in a Chinese Han population.

**Methods:**

Sixteen migraine patients with IBS and thirteen age- and gender-matched IBS patients with similar dietary and lifestyle habits were enrolled in this pilot study. Demographic data, clinical data, eating habits, lifestyle habits, comorbidities, and medications were recorded using a unified case registration form. Questionnaires for the Migraine Disability Assessment (MIDAS), Pittsburgh Sleep Quality Index (PSQI), Hamilton Anxiety Scale (HAMA), and Hamilton Depression Scale (HAMD) were completed. Fecal samples were collected, and microbial DNA was extracted. Gut microbiota 16S ribosomal RNA (16S rRNA) gene sequencing targeting the V4 region was performed using the Illumina HiSeq 2500 high-throughput sequencing platform. The relationships between gut microbiota and clinical characteristics of migraine were analyzed.

**Results:**

The structure of gut microbiota differed between migraine patients with IBS and patients with IBS, while the richness and diversity of gut microbiota in migraine patients with IBS showed no significant difference from that of patients with IBS. We found a higher relative abundance of the genus Parabacteroides and a lower relative abundance of the genera Paraprevotella, Lachnospiraceae_UCG-010, Lactococcus, Collinsella, and Comamonas in migraine patients with IBS than in patients with IBS. According to random forest predictive models, the phylum Bacteroidota shows the most important role in migraine patients with IBS. Furthermore, no statistical correlation was found between significantly different taxa at the genus level and migraine clinical data.

**Conclusion:**

This study identified that altered gut microbiota occurred in Chinese Han migraine patients with IBS, but no correlation was found between gut microbiota and the clinical characteristics of migraine. Further study is needed to better understand the role of gut microbiota in the pathogenesis of migraine in IBS.

## Introduction

Migraine is a common functional disorder characterized by recurrent headache accompanied by various autonomic, affective, and cognitive symptoms (1). Irritable bowel syndrome (IBS) is a common functional gastrointestinal disorder characterized by abdominal pain and altered bowel habits without the presence of organic lesions (2). Migraine and IBS share many similarities (3), such as incidence, female predominance, characterized by chronic and recurrent pain, lack of organic damage, similar trigger factors, benign course, and central hypersensitization. Additionally, both disorders are often associated with comorbidities such as somatic and psychiatric diseases. The mechanisms underlying this association are not entirely clear. Migraine and IBS can alter gut microbiota composition and thereby may affect the gut-brain axis and inflammatory status (3). In addition, hereditary and genetic polymorphism, serotonin, and sexual hormones are also believed to play a role (3).

However, the prognosis of IBS is fairly good, whereas that of migraine is worse since suicide and stroke are risk factors associated with migraine (4). According to the Global Burden of Disease (GBD) Study 2018 (5), migraine has become the leading cause of disability in those aged less than 50 years. Previous studies found that migraine is frequently reported in patients with IBS. A study found that approximately 17% of patients with IBS had migraine, while only 8% of the control group suffered from migraine (6). A meta-analysis of six studies showed that the risk of migraine in patients with IBS was 25–50%, while that in the control group was 4–19%, and individuals who suffered from IBS had a coexisting headache with an estimated odds ratio of approximately 2.66 (4). Migraine in patients with IBS worsens the prognosis of IBS. However, biomarkers for migraine in patients with IBS have not yet been discovered.

Previous studies have found that gut microbiota dysbiosis plays an important role in IBS (7, 8). Emerging evidence suggests that the gut microbiota also plays a role in migraine. Animal experiments by our team verified that the gut microbiome was involved in normal mechanical pain sensation and the pathogenesis of migraine (9). Another study showed that gut microbiota dysbiosis contributed to the chronicity of migraine-like pain by upregulating TNFα levels in the trigeminal nociceptive system (10). A clinical study showed that probiotics could be an effective and beneficial supplement to improve migraine headaches in those with both chronic and episodic migraines (11). Another clinical study indicated that food elimination based on IgG antibodies in migraine patients with IBS may effectively reduce symptoms associated with both disorders and has a positive impact on the quality of life in patients and on the healthcare system (12). It is currently believed that the gut microbiota may act through the microbiota–gut–brain axis, which refers to bidirectional interactions between the gut microbiome and brain *via* the vagus nerve, enteroendocrine signaling, immune system crosstalk, and neurotransmitters (13).

Recent evidence from bacterial cultures suggests that migraine patients with IBS present a higher incidence and severity of fecal dysbiosis than patients with IBS (14). However, the precise characteristics of the gut microbiota in migraine patients with IBS have not been fully elucidated. The aim of this study was to explore the composition of gut microbiota in migraine patients with IBS in a Chinese Han population.

## Materials and methods

### Subjects

**Figure 1:**
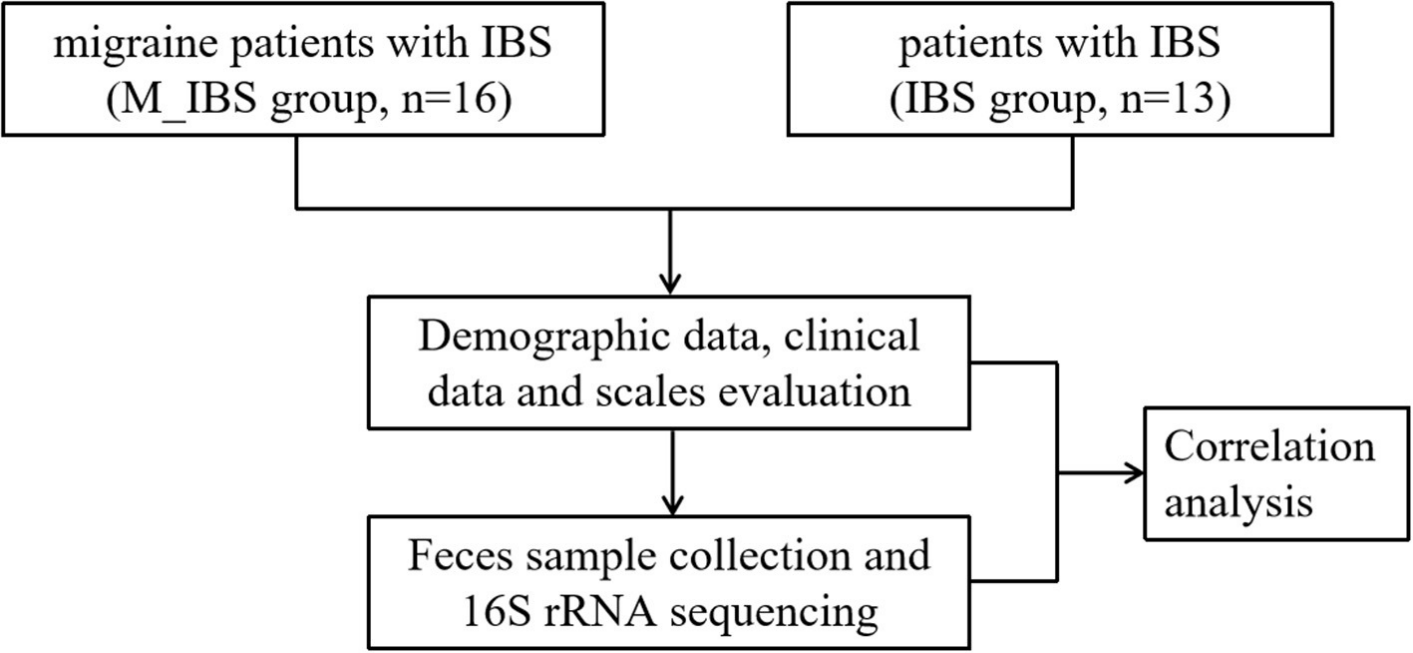
Flowchart illustrating the recruitment of migraine patients with IBS and patients with IBS and the research implementation plan.

Migraine patients with IBS were recruited at the International Headache Center of Chinese PLA General Hospital from April to August 2016. Age- and gender-matched patients with IBS were recruited from visitors coming to the Medical Examination Center for routine exams. The sample size was calculated by G^*^Power (ver. 3.1.9.7) based on the *t*-test design (15). In accordance with the Ethics Committee of PLA General Hospital, all participants were eligible for inclusion if they were aged 18–60 years and provided informed consent. This study was conducted in accordance with the guidelines set forth by the Declaration of Helsinki. The migraine diagnosis was made by experienced neurologists at the headache center, and the IBS diagnosis was made by experienced gastroenterologists. Thus, the study population comprised migraine patients with IBS (M_IBS group) and patients with IBS (IBS group) (Figure 1).

All participants met the Rome IV criteria for the diagnosis of IBS (2), and migraine was diagnosed according to the third edition of the International Headache Society classification (ICHD-3) (16).

Potential subjects with any of the following were excluded from this study: any other type of headache defined by the ICHD-3; antibiotic therapy at least 3 months before enrollment into the cohort; diarrhea on the day of fecal sampling; the score assessed using the Hamilton Rating Scale for Anxiety (HAMA) was over 21, and the score assessed using the Hamilton Rating Scale for Depression (HAMD) was over 20; any previous serious medical condition, including both somatic and psychiatric dysfunctions; drug misuse, overuse, or daily intake of medication; and pregnant or nursing females.

### Clinical data collection

Patients were interviewed for medical history. Each patient underwent a detailed physical and neurological examination and either magnetic resonance imaging or computed tomography of the head to rule out organic diseases of the brain. The following detailed information was recorded for each participant: demographic and headache data; eating habits; lifestyle habits; and comorbidities and medications. Information regarding headaches included disease duration (DD), attack frequency (AF), visual analog scale (VAS) score, and MIDAS score, which were evaluated by the migraine disability assessment (MIDAS) questionnaire (17). Sleep condition was evaluated using the Pittsburgh Sleep Quality Index (PSQI) (18), and mood condition was assessed using the HAMA (19) and HAMD (20) (Figure 1).

### Fecal sample collection and DNA extraction

The disposable sterile collection container and tubes were distributed to the participants in advance. After the feces were discharged into the sterile container, the middle part of the feces was placed in the tube using a sterile stick. Fecal samples were immediately stored in liquid nitrogen and later transferred into a −80°C freezer for preservation.

Genomic DNA in the stool samples (approximately 100 mg per sample) was extracted using a Quant-iT™ PicoGreen™ dsDNA Assay Kit (P11496, Invitrogen™, Thermo Fisher Scientific). The concentration of genomic DNA in each fecal sample was quantified using a NanoDrop 2000 spectrophotometer (Thermo Scientific, MA, USA). DNA integrity and sizes were assessed using 1% agarose gel electrophoresis (AGE).

### 16S rRNA sequencing and data processing

The gene located in the 16S rRNA V4 region was detected by specific primers, namely, 515F: GTGCCAGCMGCCGCGGTAA and 806R: GGACTACHVGGGTWTCTAAT. The NEBNext^®^ Ultra™ RNA Library Prep Kit for Illumina^®^ (E7530 L, NEB) was used to generate sequenced libraries on the Illumina HiSeq platform (Allwegene Technologies Inc., Beijing, China). The raw data were mainly processed using QIIME 2.0, USEARCH (Version 10.0.240), and other R packages mentioned below (21, 22). Trimmomatic was used to filter the nucleotides of poor quality, and reads < 50 nt were removed (parameters: LEADING: 20, TRAILING: 20, MINLEN: 50) (23). FLASH and Pear were used to assemble overlapping read pairs (24, 25). Chimeras were filtered out by UCHIME (26). The clean tags were left after the screening flow above, and they were clustered into operational taxonomic units (OTUs) by the UPARSE algorithm with a sequence similarity no less than 97% (27). Finally, an OTU table was obtained by quantifying the frequency of the OTUs in each sample. Simultaneously, the OTUs were aligned to the SILVA 132 database and assigned taxonomy at the kingdom, phylum, class, order, family, genus, and species levels (28).

### Statistical analyses

IBM SPSS Statistics for Windows, version 26.0 (IBM Corp., Armonk, N.Y., USA) and R software (ver. 3.6.1, the R Project for Statistical Computing) were used for the statistical analysis. Comparisons between groups were performed using Pearson's chi-square test for categorical variables and the Wilcoxon rank-sum test and Student's *t*-test for quantitative variables. To control the false discovery rate (FDR) for multiple testing, the q-value (corrected *p*-value) was calculated using the Benjamini–Hochberg method. Alpha diversity and beta diversity measures were calculated using the QIIME program based on the rarefied OTU counts. Differential abundance analysis was performed using the Wilcoxon rank-sum test at the phylum and genus levels. Distinguishment of the gut microbiota specific to migraine patients with IBS was identified using the linear discriminant analysis (LDA) effect size (LEfSe) method (LEfSe, https://huttenhower.sph.harvard.edu/galaxy/) (29), which is part of the QIIME package. Random forest (RF) models were used to predict disease status based on gut microbiota and the clinical data profile (significantly different taxa at each level and OTUs assessed using the Wilcoxon rank-sum test) using the default parameters of the R implementation of the algorithm (Boruta algorithm, “randomForest” package) (30). Correlations between migraine clinical data and significantly different taxa at the genus level with a prevalence ≥10% for 16 migraine patients with IBS were calculated using Spearman's rank correlation analysis with the R package “cor.test”. *P* < 0.05 was considered to be statistically significant.

## Results

### Clinical characteristics

The demographic characteristics of the M_IBS group and IBS group are shown in Table 1. The study population consisted of 29 Chinese Han people with IBS, including 16 migraine patients with IBS patients (5 men and 11 women) and 13 patients with IBS (3 men and 10 women). The age range of the participants was from 23 to 58 years. The average age of migraine patients with IBS patients was 39.69 ± 11.57 years, while that of patients with IBS was 37.00 ± 8.70 years. There was no significant difference between the two groups in sex (χ^2^ = 0.240, *P* = 0.697), age (*t* = 0.693, *P* = 0.494), BMI (*t* = 0.971, *P* = 0.340), education (χ^2^ = 1.203, *P* = 0.273), or region (χ^2^ = 1.745, *P* = 0.488). No significant difference was found in PSQI (*Z* = −0.773, *P* = 0.439) and HAMD (*t* = 2.028, *P* = 0.053) scores between the two groups, while HAMA scores were higher in the M_IBS group than in the IBS group (*t* = 2.988, *P* = 0.006). However, the HAMA and HAMD scores of all subjects did not meet the diagnostic criteria for anxiety and depression; that is, anxiety or depression was not observed in any of the subjects included in this study.

**Table 1:** Demographic characteristics in the M_IBS and IBS groups.

**Variable**	**M_IBS**	**IBS**	***p*-value**
Number, n	16	13	
Gender, n Male/n Female	5/11	3/10	0.697
Age, y	39.69 ± 11.57	37.00 ± 8.70	0.494
BMI, kg/m^2^	23.42 ± 4.09	22.07 ± 3.23	0.340
Education, *n* ≧9y/*n* >9y	5/11	1/12	0.273
Regions, n North of China /n South of China	14/2	13/0	0.488
PSQI, median (IQR)	5 (4.75)	4 (7)	0.439
HAMA	9.88 ± 4.49	4.77 ± 4.40	0.006**
HAMD	6.25 ± 4.49	3.31 ± 2.95	0.053

^**^p < 0.01; BMI, body mass index; PSQI, Pittsburgh Sleep Quality Index; IQR, interquartile range; HAMA, Hamilton Anxiety Scale; HAMD, Hamilton Depression Scale.

The eating and lifestyle habits of the M_IBS group and IBS group are shown in Table 2. There was no significant difference between the two groups in eating habits, including smoking (χ^2^ = 0.050, *P* = 1.000), alcohol (χ^2^ = 0.562, *P* = 0.632), tea (χ^2^ = 0.082, *P* = 1.000), coffee (χ^2^ = 0.738, *P* = 0.606), breakfast (χ^2^ = 0.738, *P* = 0.606), refined grain (*Z* = −0.839, *P* = 0.401), coarse grain (*Z* = −0.923, *P* = 0.356), takeaway food (χ^2^ = 0.057, *P* = 1.000), beans (χ^2^ = 0.014, *P* = 1.000), yogurt (χ^2^ = 2.644, *P* = 0.192), meat (χ^2^ = 0.842, *P* = 1.000), vegetables (χ^2^ = 0.842, *P* = 1.000), fruits (χ^2^ = 0.842, *P* = 1.000), and fermented food (χ^2^ = 0.562, *P* = 0.632), and lifestyle habits, including bowel movements (bowel movements per day χ^2^ = 1.756, *P* = 0.238; bowel movement quality χ^2^ = 4.253, *P* = 0.119), exercise (χ^2^ = 0.293, *P* = 0.588), staying up late (χ^2^ = 0.566, *P* = 0.667), pressure (χ^2^ = 0.042, *P* = 0.837), and mood (χ^2^ = 0.404, *P* = 0.663).

**Table 2:** Eating habits and lifestyle habits in the M_IBS and IBS groups.

	**M_IBS**	**IBS**	***p*-value**
Number, n	16	13	
Smoking, *n* (%)	2(12.5)	2(15.4)	1.000
Alcohol, *n* (%)	2(12.5)	3(23.1)	0.632
Tea, *n* (%)	3(18.8)	3(23.1)	1.000
Coffee, *n* (%)	3(18.8)	1(7.7)	0.606
**Breakfast per week**			0.606
< 3	3	1	
≧3	13	12	
Refined grain (Median [IQR], 50 g per day)	5 (1.75)	4(2)	0.401
Coarse grain (Median [IQR], 50 g per day)	1 (0)	1(1)	0.356
**Take away food per week**			1.000
< 3	13	11	
≧3	3	2	
**Beans per week**			1.000
< 3	12	10	
≧3	4	3	
**Yogurt per week**			0.192
< 3	16	11	
≧3	0	2	
**Meat**			1.000
Occasionally	1	0	
Regularly	15	13	
**Vegetable**			1.000
Occasionally	1	0	
Regularly	15	13	
**Fruit**			1.000
Occasionally	1	0	
Regularly	15	13	
**Fermented food per week**			0.632
< 3	14	10	
≧3	2	3	
**BM per day**			0.238
≧1	10	11	
2-3	6	2	
**BMQ**			0.119
Loose	8	3	
Normal	5	9	
Solid	3	1	
**Exercise per week**			0.588
< 3	9	6	
≧3	7	7	
**Stay up late per week**			0.667
< 3	13	9	
≧3	3	4	
Great pressure, *n* (%)	8(50)	6(46.2)	0.837
**Happy mood per week**			
< 3	4	2	0.663
≧3	12	11	

IQR, interquartile range; BM, bowel movements; BMQ, bowel movement quality (loose: tend toward diarrhea; solid: tend toward constipation).

Comorbidities and medications of the M_IBS group and IBS group are shown in Table 3. There was no significant difference between the two groups in comorbidities, including hypertension (χ^2^ = 0.023, *P* = 1.000), hyperlipidemia (χ^2^ = 2.719, *P* = 0.232), diabetes (χ^2^ = 0.842, *P* = 1.000), allergies (χ^2^ = 0.240, *P* = 0.697), asthma (χ^2^ = 0.842, *P* = 1.000), and gastric ulcer (χ^2^ = 0.023, *P* = 1.000), and medications, including antihypertensives (χ^2^ = 0.023, *P* = 1.000), statins (χ^2^ = 0.842, *P* = 1.000), antidiabetic drugs (χ^2^ = 0.842, *P* = 1.000), and nonsteroidal anti-inflammatory drugs (NSAIDs) (χ^2^ = 3.770, *P* = 0.107).

**Table 3:** Comorbidities and medications in the M_IBS and IBS groups.

	**M_IBS**	**IBS**	***p*-value**
**Comorbidities**, ***n*** **(%)**			
Hypertension	1(6.3)	1(7.7)	1.000
Hyperlipidemia	3(18.8)	0(0)	0.232
Diabetes	1(6.3)	0(0)	1.000
Allergies	5(31.3)	3(23.1)	0.697
Asthma	1(6.3)	0(0)	1.000
Gastric ulcer	1(6.3)	1(7.7)	1.000
**Medications**, ***n*** **(%)**			
Antihypertensives	1(6.3)	1(7.7)	1.000
Statins	1(6.3)	0(0)	1.000
Antidiabetic Drug	1(6.3)	0(0)	1.000
NSAIDs	4(25)	0(0)	0.107

NSAIDs, nonsteroidal anti-inflammatory drugs.

The clinical features of migraine in the M_IBS group are shown in Table 4. The median AF was 3.67 times per month, and the interquartile range (IQR) was 7.58. The average DD was 15.81 ± 11.11 years, and the average VAS score was 7.88 ± 1.19. The median MIDAS was 27, and the IQR was 63.5. The median number of MIDAS days was 11 days, and the IQR was 22.75. The average MIDAS severity was 7.88 ± 1.19.

**Table 4:** Clinical features of migraine in the M_IBS group.

**Clinical features of migraine**	**M_IBS**
**AF (Median[IQR], times per month)**	3.67(7.58)
DD, years	15.81 ± 11.11
VAS	7.88 ± 1.19
**MIDAS, Median (IQR)**	27(63.5)
MIDAS days	11(22.75)
MIDAS severity	7.88 ± 1.19

AF, attack frequency; DD, disease duration; VAS, visual analog scale; MIDAS, migraine disability assessment; IQR, interquartile range.

### Alpha and beta diversity between the M_IBS and IBS groups

**Figure 2:**
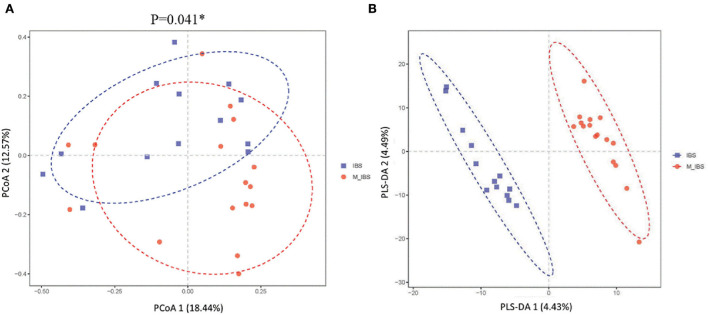
β-diversity indices of gut microbiota in the M_IBS and IBS groups. Differences in beta diversity indices between the M_IBS and IBS groups were measured using PCoA based on Bray–Curtis **(A)** and PLS-DA **(B)**, and significant *P*-values are indicated. The axes represent the two dimensions explaining the greatest proportion of variance in the communities. Each symbol represents a sample, and the points of different colors or shapes in the figure represent different groups. The scales on the horizontal and vertical axes are relative distances, while PCoA 1, PCoA 2, PLS-DA 1, and PLS-DA 2 represent the suspected influencing factors for the deviation of the microbial composition of the two groups of samples. M_IBS, migraine with irritable bowel syndrome; IBS: irritable bowel syndrome; OUT, operational taxonomic unit; PCoA, principal coordinates analysis; PLS-DA, partial least squares discrimination analysis.

Alpha diversity indices, including Chao1, observed species, phylogenetic diversity whole tree, and Shannon and Simpson indices, were analyzed to quantify species abundance and diversity based on OTU levels. There was no significant difference between the M_IBS and IBS groups in α-diversity indices (chao1: *P* = 0.487; observes_species: *P* = 0.661; PD_whole_tree: *P* = 0.358; Shannon: *P* = 0.546; Simpson: *P* = 0.408), indicating that the richness and diversity of the gut microbiota in migraine patients with IBS patients were not different from that of patients with IBS. However, significant differences were found in β-diversity based on Bray–Curtis principal coordinate analysis (PCoA; *P* = 0.041; Figure 2A) and partial least squares discrimination analysis (PLS-DA; *P* < 0.001; Figure 2B) between the M_IBS and IBS groups, which meant that the gut microbial structure in the M_IBS group was significantly different from that in the IBS group.

### Taxa alteration between the M_IBS and IBS groups

**Figure 3:**
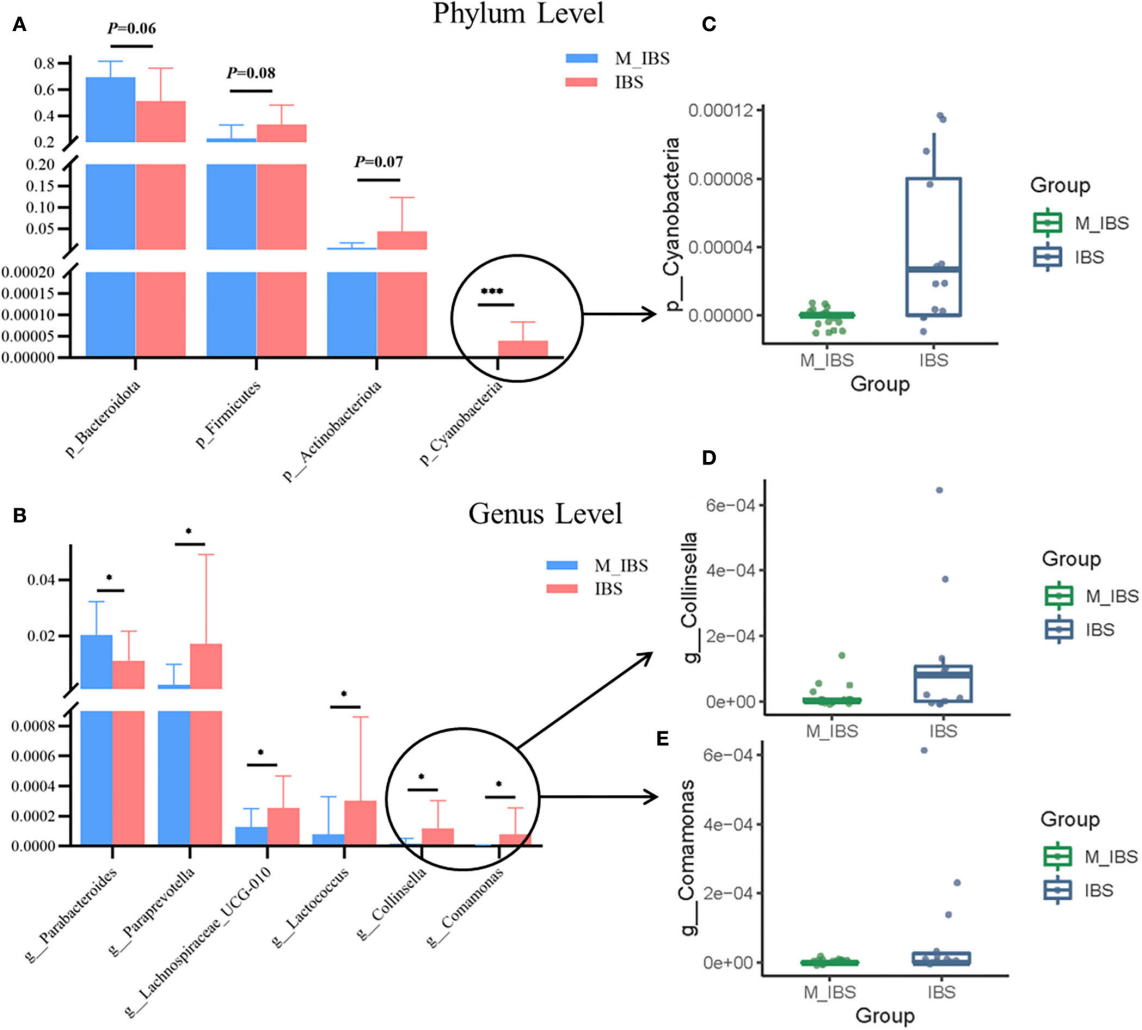
Relative abundances of gut microbiota in the M_IBS and IBS groups. **(A,B)** Bar plots comparing abundances of differentially abundant phyla and genera between the M_IBS and IBS groups, and only *p* < 0.05 or trending results are shown. **(C–E)** Box plots comparing differential phyla and genera with lower relative abundance values in bar plots between the M_IBS and IBS groups. These “signature” taxa were selected using Wilcoxon rank-sum tests and a false discovery rate of 5%. Error bars represent standard deviations, and phylum-level and genus-level taxa are plotted. *P < 0.05; ***P < 0.001. M_IBS, migraine with irritable bowel syndrome; IBS, irritable bowel syndrome.

**Figure 4:**
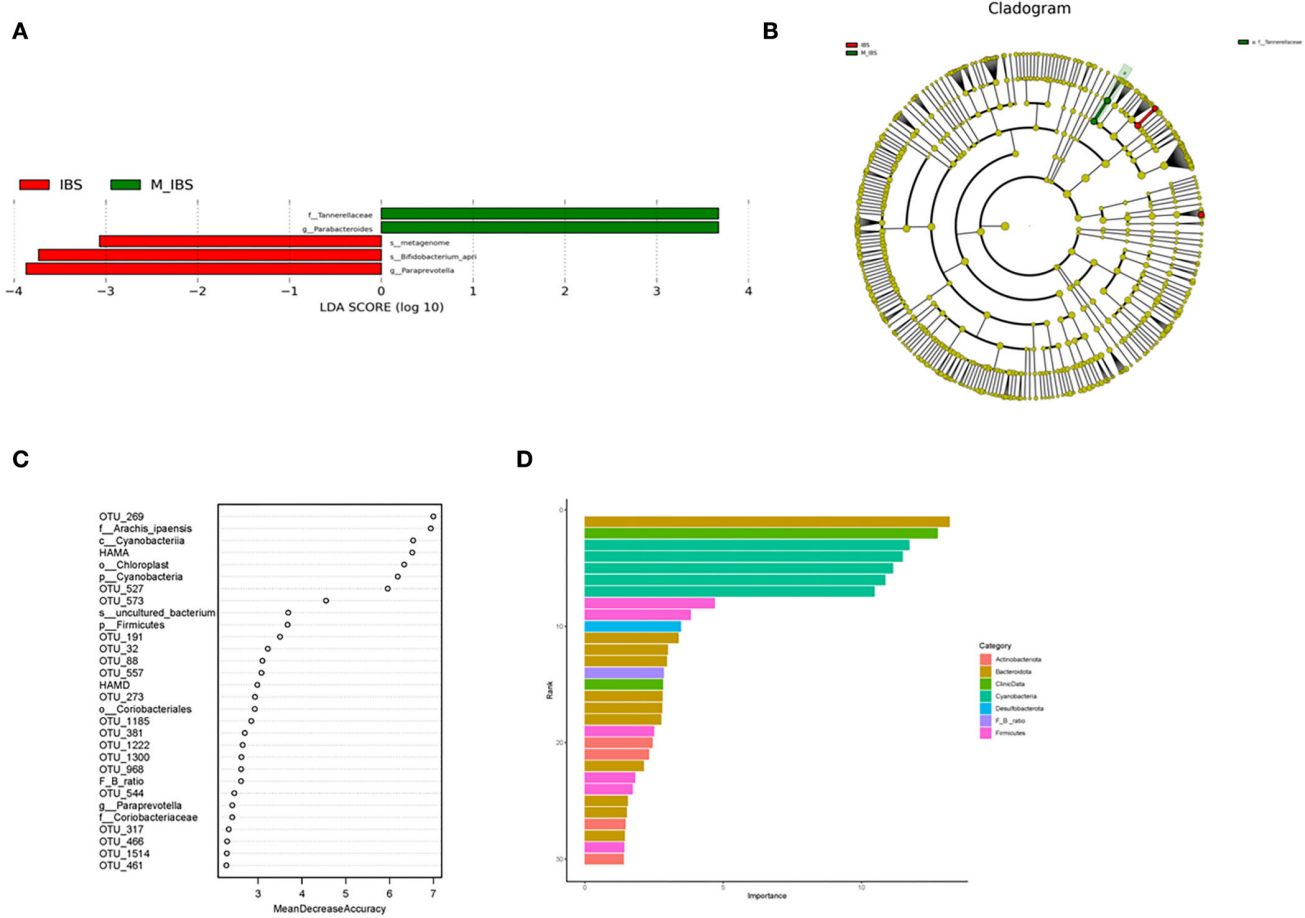
Taxonomic differences in gut microbiota in the M_IBS and IBS groups. **(A)** Linear discriminant analysis (LDA) effect size (LEfSe) analysis revealed significant taxonomic differences in gut microbiota between the M_IBS group (positive score) and the IBS group (negative score). The LDA scores (log10) >3 and *P* < 0.05 are listed. **(B)** Cladogram using the LEfSe method indicating the phylogenetic distribution of gut microbiota in the M_IBS and IBS groups. **(C)** The predictive model based on differentially abundant taxa and clinical data using an RF model. The relative importance of each index in the predictive model was determined using the mean decreasing accuracy and the Gini coefficient. **(D)** Variable importance of correlated phylum-level abundance taxa, F/B ratio, and clinical data. M_IBS, migraine with irritable bowel syndrome; IBS, irritable bowel syndrome; p, phylum; c, class; o, order; f, family; g, genus; s, species; LDA, linear discriminant analysis; LEfSe, linear discriminant analysis effect size; RF, random forest; F/B ratio, Firmicutes/Bacteroidetes ratio.

The relative abundance of the gut microbiota in the M_IBS and IBS groups at the phylum and genus levels is shown in Figure 3. Eleven phyla and 46 genera were evaluated in all subjects. We used the Wilcoxon rank-sum test to perform differential abundance analyses of differentially abundant phyla and genera between the M_IBS and IBS groups at a false discovery rate of 5%. At the phylum level, we identified a higher relative abundance of the phylum Bacteroidota (*P* = 0.056) and a lower relative abundance of the phyla Firmicutes (*P* = 0.083) and Actinobacteriota (*P* = 0.072) in the M_IBS group than in the IBS group, but the differences were not statistically significant (Figure 3A). The phylum Cyanobacteria was only found in the IBS group but not in the M_IBS group (*P* < 0.001, Figures 3A,C). At the genus level, the relative abundance of the genus Parabacteroides was higher in the M_IBS group, and the relative abundance of the genera Paraprevotella, Lachnospiraceae_UCG-010, Lactococcus, Collinsella, and Comamonas was higher in the IBS group (*P* < 0.05, Figures 3B,D,E). Differences in the taxa at the genus level are detailed in Figure 3. To identify important taxonomic differences between the M_IBS and IBS groups, we conducted linear discriminant analysis (LDA) effect size (LEfSe) analysis, and a logarithmic LDA score cutoff of 3.0 was used. We found significant abundance differences in the gut microbiota between the M_IBS and IBS groups. The relative abundance of the genus Parabacteroides was higher in the M_IBS group, while the relative abundance of the genus Paraprevotella was higher in the IBS group (LDA score (log10) > 3, *P* < 0.05, Figures 4A,B). These results indicated that migraine patients with IBS had a differential abundance of certain genera compared to that of patients with IBS.

### Random forest predictive models

To evaluate the disease status of migraine patients with IBS based on an ensemble of decision trees, we used RF to build a predictive model based on gut microbiota and clinical data profiles using the significantly different taxa at each level and OTUs from the Wilcoxon rank-sum test as the input. In these models, four phyla and the Firmicutes/Bacteroidetes ratio (F/B ratio), three classes, four orders, four families, six genera, three species, 51 OTUs, and clinical data, including HAMA and HAMD scores, predicted migraine patients with IBS using the RF model (Figure 4C). The importance of correlated phylum-level abundance taxa, F/B ratio, and clinical data is shown in Figure 4D. According to this model, the phylum Bacteroidota shows the most important role in migraine patients with IBS.

### Correlation between gut microbiota and clinical characteristics of migraine

**Figure 5:**
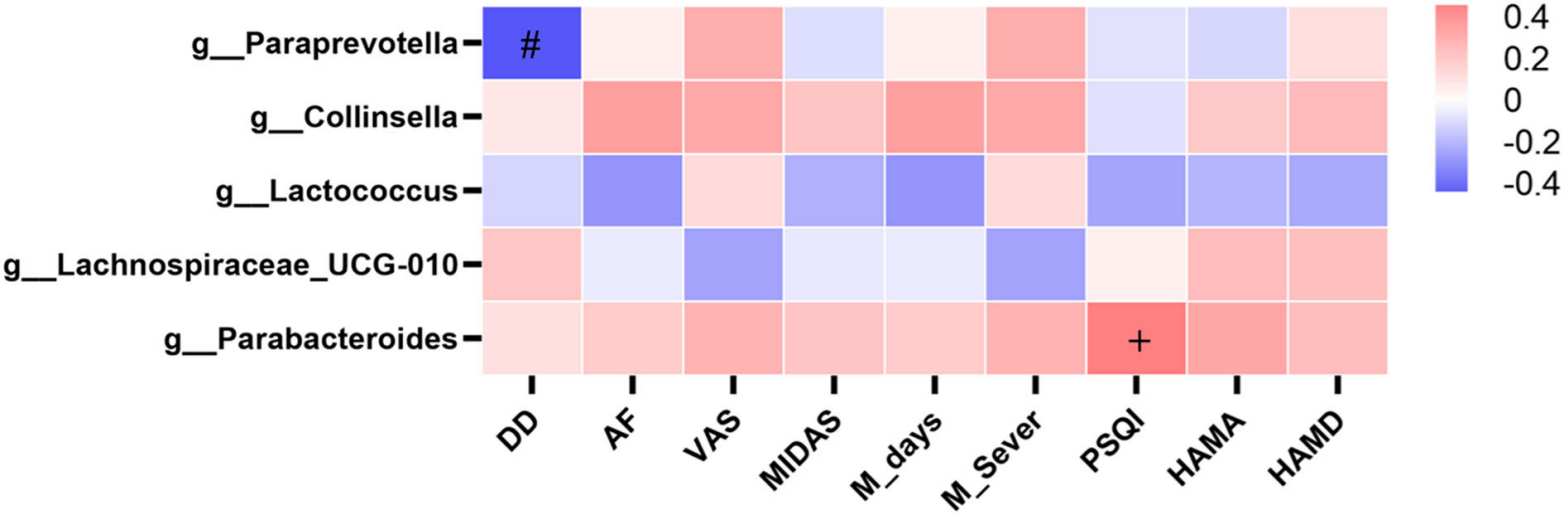
Heatmaps showing correlations between gut microbiota and clinical characteristics of migraine. Heatmap based on the abundance (sequence counts) of gut microbiota (prevalence≥10% in migraine patients with IBS) shows the correlations between significantly different taxa at the genus level and migraine clinical characteristics, including AF, DD, VAS, MIDAS, M_days, M_Sever, PSQI, HAMA, and HAMD. The intensity of the color represents the r value (correlation coefficient; negative score: blue; positive score: red). IBS, irritable bowel syndrome; AF, attack frequency; DD, disease duration; VAS, visual analog scale; MIDAS, the migraine disability assessment; M_days, MIDAS days; M_Sever, MIDAS severity; PSQI, Pittsburgh Sleep Quality Index; HAMA, Hamilton Anxiety Scale; HAMD, Hamilton Depression Scale. Spearman test, ^+^*P* = 0.056 in positive correlation, ^#^*P* = 0.075 in negative correlation.

We performed a correlation analysis between gut microbiota (significantly different taxa at the genus level, at a prevalence ≥10%) and migraine clinical data, including attack frequency (AF), disease duration (DD), pain severity (VAS), migraine disability (MIDAS), PSQI, and HAMA and HAMD scores but no statistical correlation was found (*P* > 0.05, Figure 5). The genus Parabacteroides has a possible positive correlation trend toward significance with PSQI (*r* = 0.487, *P* = 0.056), and the genus Paraprevotella has a possible negative correlation trend toward significance with DD (*r* = −0.458, *P* = 0.075) (Figure 5).

## Discussion

Migraine is frequently reported in patients with IBS, which leads to a worse prognosis for these patients; however, biomarkers for migraine in patients with IBS have not yet been discovered. In this study, we found altered gut microbiota for the first time in migraine patients with IBS in the Chinese Han population, and no differentially expressed bacterial taxa were related to the clinical characteristics of migraine. The strength of our study lies in a detailed comparison of eating habits, lifestyle habits, comorbidities, and medications, which may largely mitigate the influence of confounding factors on the results.

In our study, no significant difference was found in α-diversity indices of gut microbiota in migraine patients with IBS compared with patients with IBS, but β-diversity indices of migraine patients with IBS differed significantly from those of patients with IBS qualitatively. A metagenomic shotgun-sequencing study on gut microbiota in elderly women with migraine showed that α-diversity was evidently decreased in the migraine group at both the genus and species levels, whereas the species richness was not significantly different in the migraine and control groups at either level (31). The species richness analysis results in the previous study were consistent with our results, but the results of the α-diversity indices were not consistent with our results. We speculate that there may be several reasons for the different α-diversity results. First, the study populations are different. The subjects of our study were migraine patients with IBS, and the control group consisted of patients with IBS, while in the previous study, the subjects were elderly female migraine patients, and the control group consisted of healthy individuals. Second, stool detection methods were different. The method in our study was 16S rRNA gene sequencing, whereas the method in the previous study was metagenomic shotgun sequencing. Third, diversity analysis is based on different data. The diversity analysis in our study was based on OTUs, while the diversity analysis in the previous study was based on genus and species levels. In short, diversity analyses suggest that the structure of the gut microbiota in migraine patients with IBS is different from that of patients with IBS.

Our results showed that at the phylum level, we found a higher abundance of the gram-negative phylum Bacteroidota and a lower abundance of the gram-positive phyla Firmicutes and Actinobacteriota in migraine patients with IBS, but the differences were not statistically significant. RF predictive models also underlined the importance of the phylum Bacteroidota in migraine patients with IBS. Some studies showed similar results to ours, and decreased Firmicutes and increased Bacteroidetes in the gut microbiota were found in some central nervous system diseases, including patients with Alzheimer's disease (32), Parkinson's disease (33), multiple sclerosis (34), major depressive disorder, and autism spectrum disorder (35). However, some differences were observed between our study and previous studies. Individuals with obesity have a greater F/B ratio, more Firmicutes, and fewer Bacteroidetes (36). Additionally, patients with IBS show increased Firmicutes and decreased Bacteroidetes abundance (37). A study on the gut microbiota of patients with migraine found that elderly female patients with migraine showed significantly higher levels of Firmicutes relative to the controls (38). We speculate that changes at the phylum level may be associated with migraine in IBS. Some species within Firmicutes can produce the metabolite butyrate, a short-chain fatty acid, which predominantly plays an immunoregulatory role. All species within Bacteroidetes are gram-negative and contain the toxin lipopolysaccharide (LPS) in their outer membrane, which is known for its proinflammatory properties. The imbalance of Firmicutes and Bacteroidetes may induce an immune inflammatory response, which may be related to the pathogenesis of migraine in IBS. The phylum Cyanobacteria was only found in patients with IBS but not in migraine patients with IBS; therefore, the depletion of Cyanobacteria may be related to the occurrence of migraine in patients with IBS. However, due to its low abundance, it has not been studied extensively to date.

At the genus level, the relative abundance of Parabacteroides was higher and the abundance of Paraprevotella, Lachnospiraceae_UCG-010, Lactococcus, Collinsella, and Comamonas was lower in migraine patients with IBS. LEfSe analysis found similar results, with more Parabacteroides and less Paraprevotella in the gut microbiota of migraine patients with IBS. However, a metagenomic study on gut microbiota in elderly women with migraine showed that some detrimental species, especially Clostridium spp., were significantly enriched in migraineurs, and the controls held more beneficial microorganisms, such as Bifidobacterium adolescentis, Faecalibacterium prausnitzii, and Bacteroides intestinalis, and some “unfriendly” species, such as Odoribacter splanchnicus and Prevotella copri (31). Different results may be due to different research subjects and methods.

Parabacteroides is a group of gram-negative anaerobic bacteria in the phylum Bacteroidota that commonly colonize the gastrointestinal tract of humans. Parabacteroides exert proinflammatory effects through LPS and its metabolic end-product succinic acid (38). Paraprevotella in the phylum Bacteroidota contributes to the production of propionate by Phascolarctobacterium and then exerts an anti-inflammatory effect (39). There is limited information on the physiological role of Lachnospiraceae UCG-010 in the family Lachnospiraceae, phylum Firmicutes. Lachnospiraceae has previously been shown to be negatively correlated with new-onset, treatment-naive Crohn's disease in biopsy samples from the ileum and rectum (40). Lachnospiraceae UCG-010 increased significantly after grape powder intake for 4 weeks (41). Therefore, Lachnospiraceae UCG-010 may be a beneficial genus. Lactococcus is a genus of gram-positive facultative anaerobic bacteria in the phylum Firmicutes and is generally considered nonpathogenic toward humans in which some species produce antimicrobial compounds, such as bacteriocins, nisin, lactococcin, and recombinant proteins. Additionally, Lactococcus plays an important role in maintaining human intestinal health (42). A study found that the level of Lactococcus in the gut microbiota of nonobese patients with polycystic ovary syndrome (PCOS) was significantly lower than that of healthy controls and found that the gut microbiota changes in patients with PCOS were associated with sex hormone levels (43). Our study found that the relative abundance of Lactococcus in the gut microbiota of migraine patients with IBS was reduced, suggesting that Lactococcus may be involved in the pathophysiological process of migraine patients with IBS through changes in sex hormone levels. Comamonas in the phylum Proteobacteria is one of the few genera that can synthesize vitamin B12, which is important for normal physiological processes in humans (44). We speculate that Comamonas may be involved in the pathological process of migraine in patients with IBS through the reduction of vitamin B12 synthesis. The genus Collinsella in the phylum Actinobacteriota has been linked to proinflammatory dysbiosis in patients with type 2 diabetes (45), which is not consistent with our results. This may be due to the lower abundance of Collinsella, which is not sufficient to reverse the inflammatory effect of Parabacteroides and Paraprevotella. The changes in gut microbiota in this study suggest that migraine patients with IBS had an unhealthier gut microenvironment than patients with IBS, possibly related to inflammation, sex hormone changes, and vitamin B12 reduction.

In our study, we found no correlation between the genus in the gut microbiota and clinical characteristics of migraine, including attack frequency, disease duration, pain severity, migraine disability, sleep, anxiety, and depression. The genus Parabacteroides has a possible positive correlation trend toward significance with PSQI scores, so there may be a positive correlation between genus Parabacteroides and PSQI scores in a large sample, which means that increased Parabacteroides may be associated with poorer sleep quality. Because Parabacteroides is a proinflammatory genus (38), poor sleep quality may be associated with inflammation in the gut microbiota. The genus Paraprevotella has a possible negative correlation trend toward significance with disease duration, so there may be a negative correlation between genus Paraprevotella and disease duration in a large sample, which means that the longer the duration of migraine, the lower the abundance of Paraprevotella, and the weaker the anti-inflammatory effect of Paraprevotella (39). We speculate that prolonged migraine duration may be related to a reduction in the anti-inflammatory genus.

In this study, we explored the composition of gut microbiota in migraine patients with IBS in a Chinese Han population and found altered gut microbiota in migraine patients with IBS. However, we cannot determine whether this alteration was the result of disease progression or the cause of disease, and animal experiments are needed to verify this problem. This study may provide a new direction for the treatment of migraine patients with IBS, and further clinical research and animal experiments on probiotics or fecal bacteria transplantation will be of great help to the treatment of this disease.

The limitations should be considered. First, the sample size was limited, and studies involving a larger sample size from different populations are needed to confirm our results. Second, cohort studies will be more convincing in terms of disease progression. Third, to obtain more in-depth results, shotgun metagenome analysis can provide more detailed information in functional analysis and deeper analysis at the species level and is needed in future studies on gut microbiota in migraine patients with IBS.

## Conclusion

We find evidence for gut microbiota dysbiosis in a Chinese Han cohort of migraine patients with IBS for the first time. A well-matched control population in terms of eating habits, lifestyle habits, comorbidities, and medications is beneficial for the identification of disease-related microbiota. No correlation was found between gut microbiota and clinical characteristics of migraine. We could not clarify the detailed roles of gut microbiota in the pathogenesis of migraine in IBS from this cross-sectional study. Further studies are needed to verify whether gut microbiota can be used as a potential biomarker for migraine in patients with IBS so that novel therapeutic options aimed at regulating gut microbiota can be considered in a timely manner to improve the prognosis of migraine in IBS.

## Data availability statement

The datasets presented in this study can be found in online repositories (46). The names of the repository/repositories and accession number(s) can be found in the article/Supplementary material.

## Ethics statement

The studies involving human participants were reviewed and approved by the Ethics Committee of Chinese PLA General Hospital. The patients/participants provided their written informed consent to participate in this study.

## Author contributions

JL and WT contributed to the statistical analysis and writing of the manuscript. SY conducted the research design. ZD modified the manuscript, and all other authors contributed to collect the clinical data. All authors contributed to the article and approved the manuscript.

## Funding

This study was supported by the National Natural Science Foundation of China (grants 81901134, 81901145, and 82071226) and the Beijing Natural Science Foundation Essential Research Project (Z170002).

## Conflict of interest

The authors declare that the research was conducted in the absence of any commercial or financial relationships that could be construed as a potential conflict of interest.

## Publisher's note

All claims expressed in this article are solely those of the authors and do not necessarily represent those of their affiliated organizations, or those of the publisher, the editors and the reviewers. Any product that may be evaluated in this article, or claim that may be made by its manufacturer, is not guaranteed or endorsed by the publisher.
