# Supplementary material for: Alteration of gut microbiota in migraine patients with irritable bowel syndrome in a Chinese Han population
Source: Front Neurol. 2022 Nov 16;13:899056. doi: 10.3389/fneur.2022.899056 (PMC9709108; doi:10.3389/fneur.2022.899056)
Supplement: Supplementary file 1 [file Data_Sheet_1.docx]

Supplementary Notes：

OTU_39: k__Bacteria; p__Firmicutes; c__Clostridia; o__Oscillospirales; f__Ruminococcaceae; g__Subdoligranulum; s__uncultured_bacterium

OTU_956: k__Bacteria; p__Bacteroidota; c__Bacteroidia; o__Bacteroidales; f__Bacteroidaceae; g__Bacteroides; s__uncultured_bacterium

OTU_1029: k__Bacteria; p__Bacteroidota; c__Bacteroidia; o__Bacteroidales; f__Bacteroidaceae; g__Bacteroides; s__uncultured_organism

OTU_287: k__Bacteria; p__Actinobacteriota; c__Coriobacteriia; o__Coriobacteriales; f__Coriobacteriaceae; g__Collinsella; s__gut_metagenome

OTU_1222: k__Bacteria; p__Firmicutes; c__Clostridia; o__Lachnospirales; f__Lachnospiraceae; g__Lachnospiraceae_NK4A136_group; s__uncultured_bacterium

OTU_268: k__Bacteria; p__Bacteroidota; c__Bacteroidia; o__Bacteroidales; f__Bacteroidaceae; g__Bacteroides; s__uncultured_bacterium

OTU_573: k__Bacteria; p__Firmicutes; c__Clostridia; o__Lachnospirales; f__Lachnospiraceae; g__Lachnoclostridium; s__uncultured_bacterium

OTU_1461: k__Bacteria; p__Firmicutes; c__Clostridia; o__Lachnospirales; f__Lachnospiraceae; g__Lachnoclostridium; s__uncultured_bacterium

OTU_273: k__Bacteria; p__Firmicutes; c__Clostridia; o__Lachnospirales; f__Lachnospiraceae; g__Roseburia; s__metagenome

OTU_535: k__Bacteria; p__Bacteroidota; c__Bacteroidia; o__Bacteroidales; f__Barnesiellaceae; g__Barnesiella; s__uncultured_bacterium

OTU_1335: k__Bacteria; p__Bacteroidota; c__Bacteroidia; o__Bacteroidales; f__Bacteroidaceae; g__Bacteroides; s__uncultured_bacterium

OTU_684: k__Bacteria; p__Firmicutes; c__Clostridia; o__Lachnospirales; f__Lachnospiraceae; g__Lachnoclostridium; s__uncultured_organism

OTU_968: k__Bacteria; p__Bacteroidota; c__Bacteroidia; o__Bacteroidales; f__Bacteroidaceae; g__Bacteroides; s__uncultured_organism

OTU_184: k__Bacteria; p__Verrucomicrobiota; c__Verrucomicrobiae; o__Verrucomicrobiales; f__Akkermansiaceae; g__Akkermansia; s__uncultured_bacterium

OTU_1297: k__Bacteria; p__Firmicutes; c__Clostridia; o__Oscillospirales; f__Oscillospiraceae; g__UCG-005; s__uncultured_bacterium

OTU_281: k__Bacteria; p__Bacteroidota; c__Bacteroidia; o__Bacteroidales; f__Bacteroidaceae; g__Bacteroides; s__uncultured_bacterium

OTU_32: k__Bacteria; p__Bacteroidota; c__Bacteroidia; o__Bacteroidales; f__Bacteroidaceae; g__Bacteroides; s__uncultured_bacterium

OTU_324: k__Bacteria; p__Bacteroidota; c__Bacteroidia; o__Bacteroidales; f__Bacteroidaceae; g__Bacteroides; s__uncultured_bacterium

OTU_267: k__Bacteria; p__Proteobacteria; c__Gammaproteobacteria; o__Burkholderiales; f__Comamonadaceae; g__Comamonas; s__Comamonas_kerstersii

OTU_191: k__Bacteria; p__Desulfobacterota; c__Desulfovibrionia; o__Desulfovibrionales; f__Desulfovibrionaceae; g__Desulfovibrio; s__gut_metagenome

OTU_269: k__Bacteria; p__Bacteroidota; c__Bacteroidia; o__Bacteroidales; f__Bacteroidaceae; g__Bacteroides; s__uncultured_bacterium

OTU_1031: k__Bacteria; p__Bacteroidota; c__Bacteroidia; o__Bacteroidales; f__Bacteroidaceae; g__Bacteroides; s__uncultured_bacterium

OTU_151: k__Bacteria; p__Bacteroidota; c__Bacteroidia; o__Bacteroidales; f__Bacteroidaceae; g__Bacteroides; s__uncultured_organism

OTU_381: k__Bacteria; p__Firmicutes; c__Clostridia; o__Christensenellales; f__Christensenellaceae; g__uncultured; s__uncultured_bacterium

OTU_379: k__Bacteria; p__Bacteroidota; c__Bacteroidia; o__Bacteroidales; f__Bacteroidaceae; g__Bacteroides; s__uncultured_organism

OTU_466: k__Bacteria; p__Actinobacteriota; c__Actinobacteria; o__Bifidobacteriales; f__Bifidobacteriaceae; g__Bifidobacterium; s__Bifidobacterium_apri

OTU_407: k__Bacteria; p__Actinobacteriota; c__Actinobacteria; o__Bifidobacteriales; f__Bifidobacteriaceae; g__Bifidobacterium; s__human_gut_metagenome

OTU_1185: k__Bacteria; p__Bacteroidota; c__Bacteroidia; o__Bacteroidales; f__Bacteroidaceae; g__Bacteroides; s__uncultured_organism

OTU_1028: k__Bacteria; p__Firmicutes; c__Clostridia; o__Lachnospirales; f__Lachnospiraceae; g__Lachnospiraceae_UCG-008; s__uncultured_bacterium

OTU_317: k__Bacteria; p__Bacteroidota; c__Bacteroidia; o__Bacteroidales; f__Bacteroidaceae; g__Bacteroides; s__uncultured_bacterium

OTU_638: k__Bacteria; p__Bacteroidota; c__Bacteroidia; o__Bacteroidales; f__Bacteroidaceae; g__Bacteroides; s__Bacteroides_ovatus_CL03T12C18

OTU_557: k__Bacteria; p__Bacteroidota; c__Bacteroidia; o__Bacteroidales; f__Bacteroidaceae; g__Bacteroides; s__uncultured_organism

OTU_1118: k__Bacteria; p__Bacteroidota; c__Bacteroidia; o__Bacteroidales; f__Bacteroidaceae; g__Bacteroides; s__uncultured_bacterium

OTU_1250: k__Bacteria; p__Bacteroidota; c__Bacteroidia; o__Bacteroidales; f__Bacteroidaceae; g__Bacteroides; s__uncultured_bacterium

OTU_884: k__Bacteria; p__Proteobacteria; c__Gammaproteobacteria; o__Burkholderiales; f__Sutterellaceae; g__Parasutterella; s__uncultured_organism

OTU_1041: k__Bacteria; p__Firmicutes; c__Clostridia; o__Lachnospirales; f__Lachnospiraceae; g__Eubacterium_ventriosum_group; s__uncultured_bacterium

Raw sequence data reported in this paper have been deposited (PRJCA008682) in the

Genome Sequence Archive for Human in the Database of the National Genomics

Data Center
